# Caliver: An R package for CALIbration and VERification of forest fire gridded model outputs

**DOI:** 10.1371/journal.pone.0189419

**Published:** 2018-01-02

**Authors:** Claudia Vitolo, Francesca Di Giuseppe, Mirko D’Andrea

**Affiliations:** 1 European Centre for Medium-range Weather Forecasts, Reading, United Kingdom; 2 CIMA Research Foundation, Savona, Italy; Universidade de Vigo, SPAIN

## Abstract

The name caliver stands for CALIbration and VERification of forest fire gridded model outputs. This is a package developed for the R programming language and available under an APACHE-2 license from a public repository. In this paper we describe the functionalities of the package and give examples using publicly available datasets. Fire danger model outputs are taken from the modeling components of the European Forest Fire Information System (EFFIS) and observed burned areas from the Global Fire Emission Database (GFED). Complete documentation, including a vignette, is also available within the package.

## Introduction

Forecasting wildfires is a complex task, theoretically challenging and computationally demanding. From a theoretical point of view, fires are difficult to predict as they depend on a stochastic (unpredictable) component: the ignition. The trigger can be natural in origin like lightning and self-combustion. But it can also be due to human behavior as intentional act of arson or unintentional act of negligence. Quite commonly human-caused ignition is performed to encourage regeneration and biodiversity in the forest ecosystem or replace forest vegetation with agricultural crops [1, 2].

Once a fire is ignited, its spread, sustainability, and difficulty of control is almost exclusively determined by weather conditions [3]. Flames tend to rage out of control if certain soil and atmospheric conditions are met. As the ignition is casual and very difficult to predict, fire prediction systems, used in forest management, are design to highlight these favorable weather conditions which would allow sustained fire activity and not actual fire activities. On these premises is based one of the most widespread fire danger rating system, the Canadian forest service’s Fire Weather Index (FWI) [4, 5] which is selected here to showcase the capability of the proposed package.

The FWI is a measure of fire potential and is expressed as a numeric rating. Rating rises as fire weather becomes more severe. By construction, the relationship between the FWI numerical values and the fuel status, such as the humidity content retained in the live or dead vegetation, is weak. This implies that the same FWI values can correspond to different danger levels depending on the ecosystem. To become a meaningful tool in fire management, the FWI requires the definition of danger levels that should be site specific (we call this post-processing task ‘calibration’ hereafter). Indeed the warning levels suggested for the original FWI were derived to describe fires in a standard jack pine stand, typical of the Canadian forests. Therefore the applicability of the FWI to other parts of the planet, with vegetation characteristics dissimilar from those of the Boreal forests, requires the user to understand the fire occurrence pattern in relation to the site specific conditions. Moreover, before transferring a fire danger system from one area to another, an extensive validation/verification (the terms validation and verification are used as synonyms in this work) against forecasted and observed fire events is required to build operational confidence. In practical terms, the calibration task implies the analysis of the soil and weather conditions as synthesized by the FWI for a long period in the past, while the verification consists of the analysis of the performance of the system and danger levels applied to observed events.

From a computational point of view tailoring fire danger levels to a given area and validating the performances of an early warning system is a demanding task as it requires handling large datasets. The typical user of fire forecasting systems might not necessarily have access to powerful supercomputers or large data storages. There is a need, therefore, to design and implement post-processing algorithms in such a way that processing time and memory resources are kept to the minimum while relying on accessible hardware.

In the light of these requirements, we have developed an open-source tool called **caliver** that contains reproducible algorithms for the calibration and verification of the FWI danger levels. This is developed in the R statistical language [6], it is available from a public repository and distributed under an open license.

The calibration and verification methodologies implemented in caliver rely on the availability of long term datasets of predicted and observed fire events. In the present work we test the algorithms using the FWI predictions that the European Centre for Medium-range Weather Forecasts (ECMWF) provides to the European Forest Fire Information System (EFFIS). The modeling component of EFFIS is referred to as the Global ECMWF Fire Forecast system (GEFF, [7]). Observed fires are provided by the burned areas product from the forth generation Global Fire Emissions Database (GFED4, [8]). Both datasets are publicly available under open licenses.

In the following sections we present the caliver R package, illustrate the main functionalities and show the results of our experiments focused on calculating and validating fire danger thresholds for various areas in Europe. Europe is taken as an example study area but the methods are applicable worldwide. This work stems from the user-driven Copernicus Programme [9] which aims at developing freely and openly accessible information services based on satellite Earth Observation and in situ data. We believe that developing transparent and reproducible analysis workflows, even more if implemented within open-source initiatives is a necessary step towards the implementation of reliable modelling tools. This is because reproducible workflows aim to streamline the processing tasks as they present ready-made solutions to efficiently manipulate complex and heterogeneous datasets. Also, opening the code to the scrutiny of other experts increases the chances to implement more robust solutions and avoids duplication of efforts.

## 1 Datasets for testing

In this work, the functionalities implemented in the caliver package are tested using the data products from the Global ECMWF Fire Forecast (GEFF) system and the GFED4 dataset for observed burned areas. These datasets are described in detail in the following subsections.

### The EFFIS datasets: GEFF-reanalysis and GEFF-realtime

In the last three years fire danger forecasts have been developed at ECMWF as a part of the Copernicus Emergency Management Services under the guidance of the Joint Research Centre (JRC). A subset of GEFF data is feeding the EFFIS web portal [10]; an operational platform to access timely information for fire danger at pan European scale. 38 local and national authorities across Europe are part of the EFFIS network and have been relying on the GEFF outputs for an early identification of regions which might experience fire events due to the establishment of persistent drought conditions.

Two datasets are available for download: the GEFF-reanalysis and the GEFF-realtime. GEFF-reanalysis provides historical records of global fire danger conditions from 1980 to date. The historic record is not static but it is updated regularly following the availability of the atmospheric reanalysis dataset [11]. This dataset is used to define warning levels on the base of past events, therefore levels derived from it should not be considered static as changes in climatic conditions can alter them. GEFF-realtime provides real time deterministic high resolution and probabilistic fire danger forecasts up to 10 days ahead using weather forcings from the latest model cycle of the ECMWF weather forecast system. The real-time dataset is updated every day with a new set of forecasts. This dataset is used for operational monitoring of danger conditions.

ECMWF makes available these data products via web services: GEFF- reanalysis is already publicly available [12] and GEFF-realtime is in the process of being released [13]. The users shall be reminded that, as ECMWF’s employees, two of the authors had special access privileges to GEFF’s data products making the retrieval of the data much faster. However to facilitate access for all users, the FWI reanalysis dataset used in this paper is also accessible from a public repository [14].

Model output files are provided in NetCDF format on a regular gaussian latitude-longitude grid with longitudes ranging between 0 and 360 degrees, equivalent to -180 to +180 in geographic coordinate systems. The temporal resolution is daily and the spatial (horizontal) grid resolution is around 80 km for the reanalysis dataset based on ERA-Interim [15] and about 9 km for the realtime dataset (based on the Integrated Forecasting System model cycle CY43R1). In this work we assume GEFF-reanalysis data have already been downloaded and locally available, if not otherwise specified.

### The GFED4 dataset for observed burned areas

National inventories of wildfire activities exist in many countries but they do not have the global coverage and/or the extended record needed for a validation of a fire danger system at a global scale. Satellite observations can supply a valid alternative especially as they cover remote areas where in-situ observations are sparse. Satellite data have been used to monitor biomass burning at regional and global scales for more than two decades using algorithms that detect the radiative emission from active fires at the time of satellite overpass, and in the last decade by using burned area algorithms that directly map the spatial extent of the area affected by fires. GFED4 combines several satellite products in a homogeneous time sequence of events from 2003 onwards. Among estimations of fire emissions, it provides daily burned area fraction with a 0.25 degree spatial resolution. GFED4 combines 500 m MODIS satellite burned area maps with active fire data from the Tropical Rainfall Measuring Mission (TRMM) Visible and Infrared Scanner (VIRS) and the Along-Track Scanning Radiometer (ATSR) family of sensors.

The dataset containing GFED4 daily burned areas is used in this paper to validate the relationship between the modeled fire danger and the observed occurrence of fire episodes. The authors did not have privileged access to the dataset, just downloaded it following the instructions on the official website (www.globalfiredata.org). Data files contain 1440 columns and 720 rows and have global coverage (correspond to a horizontal resolution of 0.25 degrees) therefore before comparing burned areas with GEFF model outputs, the raster objects should be rescaled and re-sampled.

## Description of the package

### Installation and dependencies

The caliver package [16] is implemented in the R statistical language. Here we describe version 1.0, the latest stable release at the time of this writing. The package does not require compilation but depends on the following external libraries: the Climate Data Operators [17], a large tool set for working on climate and NWP model data; the NCAR Command Language [18], an interpreted language for scientific data analysis; the Geospatial Data Abstraction Library [19], a translator library for raster and vector geospatial data formats, and the NetCDF4 library [20].

Users must have the above libraries installed before attempting to install caliver. The README file (as well as the package’s home page, see link in S1 Appendix) contains a list of instructions to install dependencies on Ubuntu GNU/Linux operating systems. In the future we plan to also add installation instructions for other operating systems.

### Source code availability and documentation

Our work stands for the highest standards of scientific reproducibility from the computational point of view [21, 22]. Hence, caliver’s source code is hosted on a public repository, maintained using the git version control system and distributed under an open license: APACHE-2. Users can suggest changes and report bugs using the dedicated issue tracking system (for more information and useful links see S1 Appendix). As the package is not yet on the official CRAN repository, it can be installed using *devtools*’ [23] functionalities. Installation and load are performed as shown below:

devtools::install_github (“ecmwf/caliver”)

library (“caliver”)

The functions available in this code release are divided into four groups: retrieval, utilities, analysis and visualization. Each function is described in detail in the section on applications. The code to reproduce the content of this article is available as vignette of the package under the title ‘An introduction to the caliver package’. Additional documentation is available in the form of help pages.

### Continuous integration and unit tests

In order to have a reliable development process, system dependencies, installation and basic functionalities are tested using the travis-CI for continuous integration on a unix-based system. Unit tests for the main functions are developed using the testthat framework [24]. Software metrics, in terms of code coverage status, are tracked using the codecov platform (https://codecov.io/). The current release has a code coverage of 94%.

## Applications

The primary goal of the caliver package is to streamline the post-processing of GEFF model outputs and make the scientific workflow easily reproducible. For this reason, this section provides example applications in the form of short workflows. These are ordered chronologically, based on the sequential steps a modeller would perform to retrieve and visually explore information, calibrate fire danger levels and validate them. This often results in an increasing level of complexity. To provide a concise description, each workflow is not a stand alone exercise but the result of one workflow is often used as input in the following ones. In the code snippets, we mention functions belonging to packages other than caliver and base, using the convention package_name::function_name.

### Data retrieval

#### Get data from the fourth-generation Global Fire Emissions Database (GFED4)

Observed burned areas around the world are collected by the GFED4 in hdf format. This information is very important as it represents the ground truth for fire models, it is needed to make comparisons with reanalysis and forecast models and estimate their reliability. The function get_gfed4 allows to access the ftp server and download the data directly from an R console. Available input arguments and options are summarised in Table 1.

**Table 1:** Summary table of input arguments and options related to the function get_gfed4.

Argument	Type and description	Example usage
*start*_*date*	String. First date to download.	*start*_*date* = “2003-01-01”
*end*_*date*	String. Last date to download.	*end*_*date* = “2015-12-31”
*temporal*_*resolution*	String. Temporal resolution, the options are: *daily* (default), monthly	*temporal*_*resolution* = “daily”
*varname*	String. Variable to extract, the options are: *BasisRegions*, *BurnedArea*, *BurnedAreaUncertainty*, *MeanBurnDateUncertainty*, *source*, *TreeCoverDist*, *LandCoverDist* or PeatFraction . See GFED4 documentation for further details.	*varname* = “BurnedArea”
*region*	String. Region of interest (this only works if varname = “BasisRegions”), the options are: *GLOB* (Global), *BONA* (Boreal North America), *TENA* (Temperate North America), *CEAM* (Central America), *NHSA* (Northern Hemisphere South America), *SHSA* (Southern Hemisphere South America), *EURO* (Europe), *MIDE* (Middle East), *NHAF* (Northern Hemisphere Africa), *SHAF* (Southern Hemisphere Africa), *BOAS* (Boreal Asia), *CEAS* (Central Asia), *SEAS* (Southeast Asia), *EQAS* (Equatorial Asia), *AUST* (Australia and New Zealand)	*region* = “EURO”

The code below shows how to download daily burned area maps from 2003 to 2015, extract the variable of interest using the argument varname, and merge all the rasters into a single RasterBrick (a 3-dimensional spatial class defined in the raster package [25]). In order to carry out raster operations, this RasterBrick might need to be resampled to match the attributes of lower/higher resolution rasters. This can be done using the function raster::resample.

BurnedAreas <- get_gfed4 (start_date = “2003-01-01”,

            end_date = “2015-12-31”,

            temporal_resolution = “daily”,

            varname = “BurnedArea”,

            region = “GLOB”)

The input arguments allow to overwrite default behaviours. For instance, in the example above the temporal_resolution is set to download daily maps. Monthly maps are also available and can be downloaded by setting temporal_resolution = “monthly”.

In addition to burned areas, GFED4 also provides, as ancillary data, a map of 14 basis regions used to evaluate regional annual emission estimates. This map can be retrieved as SpatialPolygonsDataFrame (a spatial class defined in the sp package) setting varname = “BasisRegions”. By default all the basis regions are returned but the argument region can be used to extract a single region, as shown in the second example below for Europe.

BasisRegions <- get_gfed4 (varname = “BasisRegions”)

Europe <- get_gfed4 (varname = “BasisRegions”, region = “EURO”)

The reader is warned that the retrieval of burned areas and other variables from the GFED4 database should be run in the console because some of the dependencies do not currently work in all the Interactive Development Environments (IDEs, i.e. RStudio). All the other uses of the function get_gfed4 (e.g. for retrieving basis regions) and all the other functions in the caliver package can be used in IDEs.

#### Get administrative boundaries

In the following sections we make use of administrative boundaries to define danger classes for different domains. For a matter of consistency, we use the GADM database of Global Administrative Areas (http://www.gadm.org/). These areas are available as SpatialPolygonDataFrame (amongst other formats) and can be retrieved using the function raster::getData.

Although this is not a functionality built in the caliver package, we provide below the code to get polygons for the UK, Spain and Italy as well as some Italian regions and the Province of Genoa. These polygons will be used in the following sections to define danger classes at country, regional and province level. In the raster::getData function, there are three important input arguments: name (the name of the database), country (the name of the country), and the administrative level. As a general rule, level 0 corresponds to the country borders, level 1 to regions, level 2 to provinces and level 3 to local authorities. For some countries, however, this classification might be slightly different, therefore we suggest the reader to consult the GADM website for more information.

# United Kingdom

UnitedK <- raster::getData (name = “GADM”, country = “United Kingdom”, level = 0)

# Spain

Spain <- raster::getData (name = “GADM”, country = “Spain”, level = 0)

# Italy

Italy <- raster::getData (name = “GADM”, country = “Italy”, level = 0)

# Italian regions

Italy1 <- raster::getData (name = “GADM”, country = “Italy”, level = 1)

# Get polygons for Liguria, Calabria and Sicily

Liguria <- Italy1 [9,]

Calabria <- Italy1 [4,]

Sicily <- Italy1 [15,]

# Get polygon for the Province of Genoa

Italy2 <- raster::getData (name = “GADM”, country = “Italy”, level = 2)

Genoa <- Italy2 [42,]

#### Get the JRC’s fuel model map

Vegetation is the fuel for fire. In order to carry out an accurate estimation of fire danger worldwide it is important to use an homogeneous classification of land use and vegetation. Such a map was compiled by the Joint Research Centre merging information from the Global Land Cover 2000 database and regional products for Africa, Asia, and Europe [7]. Within the caliver package, a cached version of this map (called fuelmodel map) is available in the ‘inst/extdata’ folder. Fire danger indices can be masked using the fuelmodel map, removing the areas characterised by water, barren, marsh, snow and ice, urbanization, agriculture as well as no data (codes 21-27). This is achieved using the function mask_with_fuelmodel. This function accepts only one argument: the raster object to be masked.

### Utilities

#### Importing and decompressing data files

When users download GEFF data from the web interface, they obtain a Tape ARchive (.tar file) containing a single compressed file (i.e. .gz) for each day, origin and variable. This is necessary to optimise transfer of large datasets and local storage space. If data for a short time period is needed, the function import_GEFF_data_from_tar can be used to open the tar archive, decompress the gz files, merge the layers into a RasterStack and convert this to a RasterBrick object for more efficient computation. The input argument is briefly described in Table 2, while a sample dataset (geff5.tar) is available in the testdata folder of the package.

**Table 2:** Summary table of input arguments and options related to the function import_GEFF_data_from_tar.

Argument	Type and description	Example usage
*archive*	String. Path to the tar file downloaded from the GEFF web portal.	*archive* = “geff5.tar”

geff5tar <- system.file (file.path(“testdata”, “geff5.tar”),

          package = “caliver”)

b <- import_geff_data_from_tar (archive = geff5tar)

When numerous time steps for multiple variables are needed, the amount of data to retrieve can easily exceed few gigabytes. Network transfer can become the bottleneck and retrieval may be more efficient if carried out into chunks. The function utils::untar can be used to remove the first layer of compression on the data chunks. The second layer of compression can be removed using the function R.utils::gunzip. This function only decompresses one archive at the time, as an alternative users can use caliver’s decompress_gz function to decompress multiple archives simultaneously. By default, the function will look into the working directory for archives to decompress but this can be changed using the argument input_dir to specify a different directory. In the example below, for instance, input_dir points to a subfolder of the working directory called ‘tmp’. Compressed archives are removed once the information has been extracted (from .nc.gz to .nc). The input argument is briefly described in Table 3.

**Table 3:** Summary table of input arguments and options related to the function decompress_gz.

Argument	Type and description	Example usage
*input*_*dir*	String. Path to the directory storing the files to be read.	*input*_*dir* = “./tmp”

decompress_gz(input dir = “./tmp”)

#### Merge multiple files over the time dimension

If GEFF data is retrieved in chunks, the user is encouraged to store all the files in the same directory (one file per day) so that they can be merged over the time dimension using the function stack_netcdf_files. This function is a simple wrapper to the cat function of the cdo library and its input arguments are briefly described in Table 4.

**Table 4:** Summary table of input arguments and options related to the function stack_netcdf_files.

Argument	Type and description	Example usage
*input*_*dir*	String. Path to the directory storing the files to be read.	*input*_*dir* = “/home/user/in”
*varname*	String. Variable to extract.	*varname* = “fwi”
*pattern*	String. Regular expression pattern to select a subset of files.	*pattern* = “geff_reanalysis”
*recursive*	Logical. If TRUE the search is carried out on folders and subfolders, if FALSE (default) the search is carried out only in the specified folder.	*recursive* = TRUE
*output*_*file*	String. Output file path (if different from the working directory).	*output*_*file* = “FWI.nc”

The function assumes that the files are already ordered based on time, for example using a consistent file naming convention. This saves processing resources as files do not need to be opened all at the same time to be sorted over the time dimension (as in the mergetime function of the cdo library). For large volume of data this method is much faster than looping through the files and stacking them in a RasterStack.

To give an indication of the volume of data that can be handled efficiently and the average processing time, the daily FWI was extracted from the GEFF reanalysis dataset from 1980 to 2016 and downloaded in a directory called ‘tmp’. In the example below all the FWI data was merged generating a 6.6 GB output file in about 1.5 minutes using the hardware and software described in S1 Appendix.

processingTime <- system.time ({

 stack_netcdf_files (input dir = “./tmp”, output file = “FWI.nc”)

})

GEFF output files store one variable per file but this might change in the future. To make the function future-proof we have introduced the argument varname to extract only the variable of interest from multiple files. By default this function tries to merge all the NetCDF files in the input_dir folder. The function accepts regular expression patterns to select a subset of files via the argument pattern, also the search can be expanded using a recursive approach (setting the argument recursive = TRUE). The output file is named ‘output.nc’ and saved in the working directory (if not otherwise specified by the argument output_file).

### Analysis and visualisation

#### Generate maps of percentiles

GEFF reanalysis files are characterized by three dimensions (latitude, longitude and time) and a variable for each index of interest. For each index, calculating relevant quantiles cell by cell (over time) gives an indication of the local distribution of fire danger values. Such a map can be generated using the function get_percentile_raster (see Table 5 for information on the input arguments).

**Table 5:** Summary table of input arguments and options related to the function get_percentile_raster.

Argument	Type and description	Example usage
*probs*	Numeric. This is the vector of percentiles (values in the range [0,100]) to be calculated.	*probs* = c(50, 90)
*r*	Raster* object (either a RasterStack or a RasterBrick).	*r* = b
*input*_*file*	String. Path to the file containing the temporal stack of fire indices. This is usually the file generated by stack_netcdf_files.	*input*_*file* = “./inFilePath.nc”
*output*_*dir*	String. Path to the directory where files are saved. By default this is the working directory.	*output*_*dir* = “/home/user/out”

**Fig 1:**
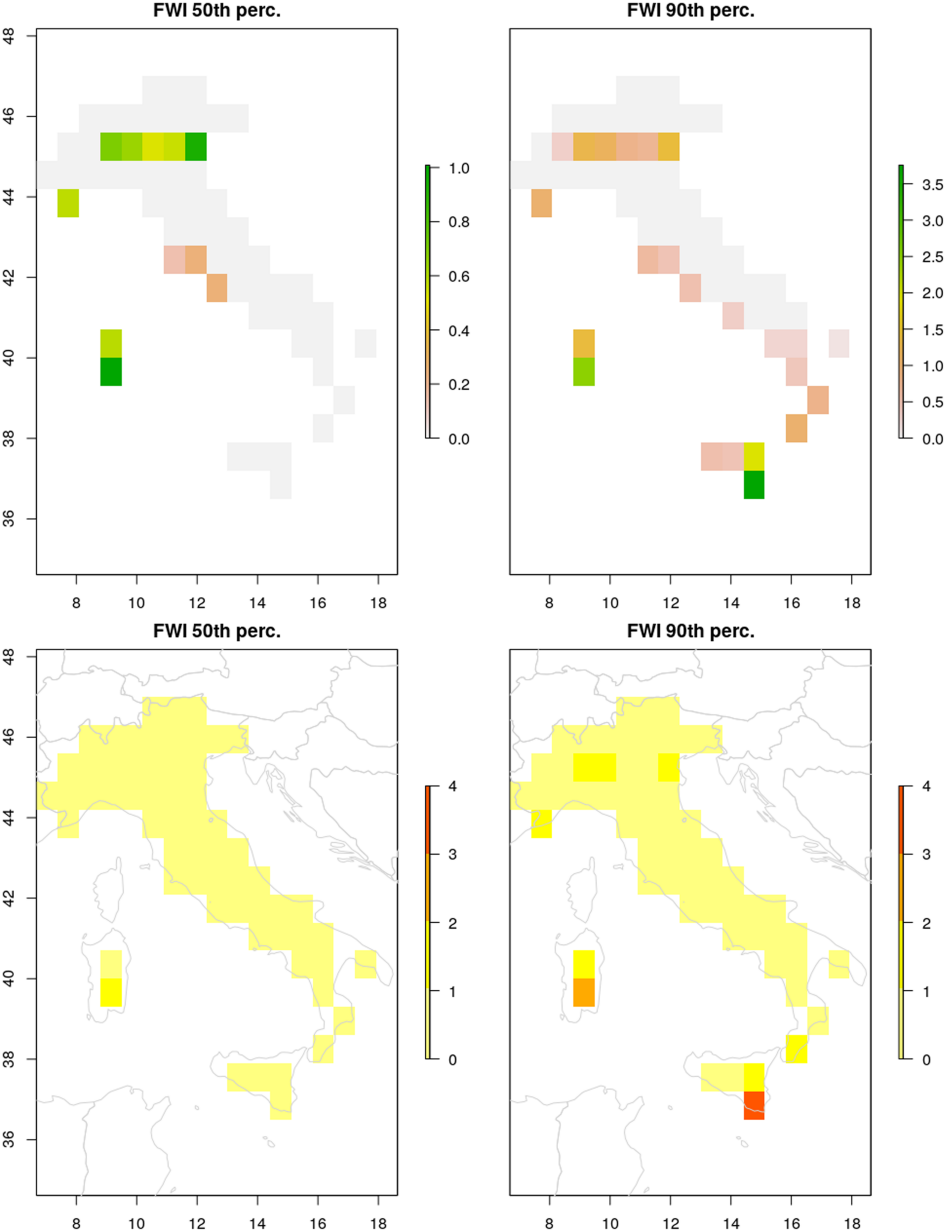
Comparison between raster plot method (top) and the caliver plot_percentile_raster function (bottom).

The example below uses the FWI.nc file (generated in the previous section) to produce a map corresponding to the 50th percentile which can be plotted using the function plot_percentile_raster, as it is described in the section ‘Plot maps of percentiles’ and shown in Fig 1.

map <- get_percentile_raster (input_file = “FWI.nc”, probs = 50)

As an alternative, the same function can be used to get percentiles from a raster brick in memory. The example below produces a list of three maps: the 50th, 75th and 90th percentiles from the object b generated in the section ‘Importing and decompressing data files’.

maps <- get_percentile_raster (r = b, probs = c(50, 75, 90))

#### Mask, crop and subset

Fire indices can be masked and cropped to match a user-defined extent, this can be a geographical bounding box or a spatial polygon such as a given administrative boundary. The indices can also be subsetted over the layer index or the time dimension (e.g. to take into account only fire seasons). The function mask_crop_subset wraps the functions raster::mask, raster::crop and raster::subset and converts the result into a RasterLayer (single layer) or RasterBrick (multiple layers). Table 6 provides a description of the input arguments, while the example below shows how to mask and crop the previously generated percentile maps over Europe, subsetting only the 50th and 90th percentiles.

**Table 6:** Summary table of input arguments and options related to the function mask_crop_subset.

Argument	Type and description	Example usage
*r*	Raster* object (either a RasterStack or a RasterBrick).	*r* = maps
*p*	SpatialPolygon* object.	*p* = Europe
*mask*	Logical. If TRUE (default) a mask is applied using *p*, if FALSE no mask is applied.	*mask* = TRUE
*crop*	Logical. If TRUE (default) the Raster* object *r* is cropped along *p*, if FALSE the object is not cropped.	*crop* = TRUE
*idx*	Numeric. Vector of indices to subset (usually this refers to the time dimension).	*idx* = 1:10

mapItaly <- mask_crop_subset (r = maps, p = Italy, idx = c(1, 3))

#### Plot maps of percentiles

The raster package provides convenient methods to plot many types of GIS layers. The caliver package builds upon these functionalities to generate pre-styled plots. The previously generated percentile maps could be printed using the raster::plot method but the map would need to be further manipulated to: rotate the longitudinal coordinates (optional), overlay a background map and set the color scale to the same range so that multiple maps become comparable. The rotation issue is due to an ambiguity in the WGS84 coordinate system (EPSG: 4326) which does not explicitly define the longitude range neither the location of the central meridian. As a consequence different applications have developed different standards: global climate and derived models (such as GEFF) set the longitude range between 0 and 360 degrees, while others between -180 and +180 degrees (e.g. GADM and GFED4).

By convention, all the functions in caliver assume WGS84 (European-centric, with longitudes ranging between -180 and +180) as the reference coordinate system to make data compatible with other sources of information. This also means that GEFF grids should be rotated prior to using the functions in this package. The rotation can be obtained in, at least, two ways: using the raster::rotate function or sellonlatbox from the cdo library. The caliver function plot_percentile_raster (see Table 7) performs these manipulations behind the scenes, also incorporating a background map and placing multiple plots in a grid-like layout with the same color scale. In the example below and in Fig 1 we compare direct outputs using raster::plot (top) and plot_percentile_raster (bottom).

**Table 7:** Summary table of input arguments and options related to the function plot_percentile_raster.

Argument	Type and description	Example usage
*maps*	Raster* object. This is usually the result of get_percentile_raster.	*maps* = maps
*rotate*_*map*	Logical. FALSE by default. If TRUE the maps are rotated from a range [0, 360] to the range [-180,+180].	*rotate*_*map* = *TRUE*
*region*	String. This is the region of interest, same argument as in *get*_*gfed*4. The options are: *GLOB* (Global), *BONA* (Boreal North America), *TENA* (Temperate North America), *CEAM* (Central America), *NHSA* (Northern Hemisphere South America), *SHSA* (Southern Hemisphere South America), *EURO* (Europe), *MIDE* (Middle East), *NHAF* (Northern Hemisphere Africa), *SHAF* (Southern Hemisphere Africa), *BOAS* (Boreal Asia), *CEAS* (Central Asia), *SEAS* (Southeast Asia), *EQAS* (Equatorial Asia), *AUST* (Australia and New Zealand)	*region*= “EURO”
…	additional graphical parameters inherited from the plot method in the raster package.	*main* = “Title”

# Use the raster plot method

raster::plot (mapItaly, main = c(“FWI 50th perc.”, “FWI 90th perc.”))

# Use the caliver plot_percentile_raster function

plot_percentile_raster (maps = mapItaly, main = c(“FWI 50th perc.”, “FWI 90th perc.”))

#### Fire danger levels

GEFF reanalysis data can be used to identify significant thresholds in fire danger levels. Danger levels can be calculated based on some user-defined parameters: the spatial extent of the area of interest and the fire season. The fire season is defined as the dry season, a period in which there is a reduced soil moisture and precipitation. In this work we adopt a convention: the fire season falls between 1st April and 31st October in the northern hemisphere, and between 1st October and 30th April in the southern hemisphere. This convention is coded in the function get_fire_season (see Table 8), which accepts at least two arguments: dates (the sequence of daily dates for which reanalysis data is available) and zone (which can be either ‘north’ or ‘south’ emisphere). There are also two optional arguments that allow to define an ad-hoc fire season: fss (which stands for Fire Season Start) and fse (which stands for Fire Season End).

**Table 8:** Summary table of input arguments and options related to the function get_fire_season.

Argument	Type and description	Example usage
*dates*	Date, sequence of daily dates	*dates* = seq.Date(from = as.Date(“1980-01-01”), to = as.Date(“2016-12-31”), by = “day”)
*fss*	Date, Fire Season Start. This date in the format Y-m-d	*fss* = as.Date(“2012-04-01”, format = “%Y-%m-%d”)
*fse*	Date, Fire Season End (date in the format Y-m-d)	*fse* = as.Date(“2012-10-31”, format = “%Y-%m-%d”)
*zone*	String, this can either be *north* (default) or *south*.	*zone* = “north”

# Define period for reanalysis

dataDates <- seq.Date(from = as.Date(“1980-01-01”),

          to = as.Date(“2016-12-31”),

          by = “day”)

# Define a function to extract fire seasons in Europe

seasons <- get_fire_season(dates = dataDates, zone = “north”)

# Create an index of fire season dates

fireSeasonIndex <- which(seasons == TRUE)

In order to generate danger levels for Europe (although it could be used anywhere), we used the fire season defined above to subset the FWI dataset spanning the period 1980-2016 (daily time step) cropped and masked over the European border. We assume that the extreme value for FWI corresponds to the median of the yearly 98th percentile (related to the number of days a fire is expected to occur in a year) of the FWI subset. The yearly FWI extremes are then transformed into fire intensity (*I*), according to equations 32 in [5].

$$\begin{array}{c} ln\left(0.289I\right)=0.980{\left[ln\left(FWI\right)\right]}^{1.546} \end{array}$$ (1)

The other classes are then arranged on a geometrical progression in terms of the fire intensity by using a constant ratio, *r*, between two consecutive classes. The *I* values are then converted back into FWI using Eq 2 (equation 31 in [5]).

$$\begin{array}{c} ln\left(FWI\right)=1.013{\left[ln\left(0.289I\right)\right]}^{0.647} \end{array}$$ (2)

The choice of using the fire intensity to define the danger classes and not directly FWI is dictated by the fact that there is a simple exponential relationship between *I* and the severity of fire conditions. The algorithm to calculate danger levels is coded in the function get_fire_danger_levels. As shown in Table 9, this function accepts two input arguments: the fire_index (i.e. the FWI subset generated previously) and ndays (the number of days per year in which a fire is expected to occur, 4 by default). In the example below the danger levels for Europe are calculated. The result is a numeric vector: 2, 5, 10, 19, 33. These numbers identify 6 classes of danger: very low (*FWI* < 2), low (2 ≥ *FWI* > 5), moderate (5 ≥ *FWI* > 10), high (10 ≥ *FWI* > 19), very high (19 ≥ *FWI* > 33) and extreme (*FWI* ≥ 33).

**Table 9:** Summary table of input arguments and options related to the function get_fire_danger_levels.

Argument	Type and description	Example usage
*fire*_*index*	RasterBrick. This contains the fire index to calculate the thresholds for. Please note that *names*(*fire*_*index*) should contain dates.	*fire*_*index* = FWI
*ndays*	Numeric. Number of days per year in which a fire is expected to occur. By default this is 4 days.	*ndays* = 4

# Load FWI dataset obtained previously

FWI <- raster::brick (“FWI.nc”)

# Mask/Crop/Subset FWI over Europe

FWIEURO <- mask_crop_subset (r = FWI, p = Europe, idx = fireSeasonIndex)

# Calculate levels

EuropeThr <- get_fire_danger_levels(fire index = FWIEURO)

The same procedure can be repeated for any spatial extent, with a minimum area equal to the FWI cell size. The vignette ‘An introduction to the caliver package’ contains a concise script to calculate danger levels for all the countries in the European Union. In the examples below, instead, we show how to calculate the danger levels for few countries (i.e. Italy, United Kingdom and Spain) as well as at regional (Liguria Region, Calabria Region and Sicily) and province level (Province of Genoa, part of Liguria Region). All the thresholds calculated, along with those currently used by EFFIS are summarised in Table 10.

**Table 10:** FWI danger levels for selected areas. Values in bold are used for validation. The first row refers to thresholds defined by EFFIS, the remaining rows list the levels defined by caliver.

Area of interest	Low	Moderate	High	Very high	Extreme
Europe (EFFIS—current standard)	5.2	11.2	**21.3**	38	50
Europe	2	5	**10**	19	33
United Kingdom	1	3	**7**	12	19
Spain	2	7	**15**	30	55
Italy	2	6	**12**	23	42
Calabria Region (IT)	2	6	**12**	24	42
Sicily (IT)	2	6	**14**	27	49
Liguria Region (IT)	2	4	**9**	16	28
Province of Genoa (Liguria Region, IT)	2	4	**9**	17	29

# Mask/Crop/Subset FWI and generate thresholds for Italy

FWIIT <- mask_crop_subset (r = FWI, p = Italy, idx = fireSeasonIndex)

ItalyThr <- get_fire_danger_levels (fire_index = FWIIT)

# Repeat for other countries …

# Mask/Crop/Subset FWI and generate thresholds for Liguria Region

FWILIG <- mask_crop_subset (r = FWI, p = Liguria, idx = fireSeasonIndex)

LIGThr <- get_fire_danger_levels (fire_index = FWILIG)

# Repeat for other regions …

# Mask/Crop/Subset FWI and generate thresholds for the province of Genoa

FWIGEN <- mask_crop_subset (r = FWI, p = Genoa, idx = fireSeasonIndex)

GENThr <- get_fire_danger_levels(fire_index = FWIGEN)

As expected, the thresholds for Europe are a sort of average if compared with the thresholds of individual countries, being higher than the thresholds calculated for countries in northern Europe (i.e. United Kingdom) and lower than the thresholds for a typical country at high risk of fire, such as Italy and Spain (or other Mediterranean countries). The same applies when moving from national to regional level in Italy, where southern (warmer) regions such as Sicily and Calabria have higher risk thresholds that regions in the north of the Italian peninsula, such as Liguria. Thresholds at regional and province level are, instead, very similar because the spatial resolution of GEFF’s outputs (about 80 km cell size) is too coarse to explain the local spatial variability. This limitation will be hopefully overcome in the near future with the introduction of new reanalysis products with higher spatial resolution (i.e. ECMWF ERA-5).

#### Validate fire danger thresholds

Fire danger levels have been identified using reanalysis data, as they hold the historical information on the state of the soil and atmosphere that could generate a dangerous fire in case of ignition. Operationally, these levels are going to be used with fire forecasts to issue alerts. Validating the fire danger thresholds corresponds to check whether the forecasted fire danger signal is clearly detectable. At the time of this writing GEFF-realtime is not in the public domain yet, therefore for illustrative purpose, the example below illustrates the validation methodology using GEFF-reanalysis.

We compared observed large fires (with burned area above 50 hectares) with the corresponding reanalysis value and danger levels (looking at thresholds defined by both EFFIS and caliver). The goal is to understand whether caliver’s methodology introduces an improvement in the probability of detection.

The methodology is as follows:

1. Take the full dataset of observations (burned areas measured in hectares, available from 2003 to 2015).
2. A binary value is assigned to each cell: 1 if the burned area is greater than or equal to 50 hectares (only large fires are taken into account), 0 otherwise.
3. Mask and crop the observations over the region of interest.
4. Subset the fire index (FWI) dataset on the period 2003-2015.
5. Mask and crop the fire index over the region of interest.
6. A binary value is assigned to each cell: 1 if the fire index is greater than or equal to the high danger level, 0 otherwise.
7. If the observations have higher resolution than the fire index, the former are resampled to match the resolution of the latter using the nearest-neighbor resampling technique.
8. Count hits (cells in which observation and fire index are both equal to 1) and misses (cells in which observation is 1 and fire index is 0) and calculate the probability of detection.

The procedure illustrated in points 1 to 8 is demonstrated in the code below, where the output of function validate_fire_danger_levels (see Table 11 for details on the input arguments) is a list containing two vectors: predictions (pred) and observations (obs). These two vectors can be used to generate a contingency table summarizing the number of hits and misses and validate the high danger threshold.

**Table 11:** Summary table of input arguments and options related to the function validate_fire_danger_levels.

Argument	Type and description	Example usage
*fire*_*index*	RasterBrick. This contains the fire index (only one variable).	*fire*_*index* = FWI
*observation*	RasterBrick. This contains the observation (only one variable).	*observation* = BA
*fire*_*threshold*	Numeric. Threshold to use to select relevant fire indices.	*fire*_*threshold* = 21.3
*obs*_*threshold*	Numeric. Threshold to use to select relevant observations.	*obs*_*threshold* = 50

# If observations layers have no date, assign it!

names(BurnedAreas) <- seq.Date (from = as.Date(“2003-01-01”),

               to = as.Date(“2015-12-31”),

               by = “day”)

# Mask and crop burned areas over Europe

BA <- mask_crop_subset (r = BurnedAreas, p = Europe, mask = T, crop = T)

# For the validation we do not want to subset over the fire season

FWIEURO <- mask_crop_subset (r = FWI, p = Europe, mask = T, crop = T)

# Get predictions and observations for Europe, using EFFIS

x <- validate_fire_danger_levels (fire_index = FWIEURO,

              observation = BA,

              fire_threshold = 21.3,

              obs_threshold = 50)

# Contingency table

tab <- table (pred = x$pred, obs = x$obs)

hits <- tab [2, 2]

misses <- tab [1, 2]

# Probability Of Detection (POD)

POD <- round (hits/(hits+misses), 2) * 100

**Fig 2:**
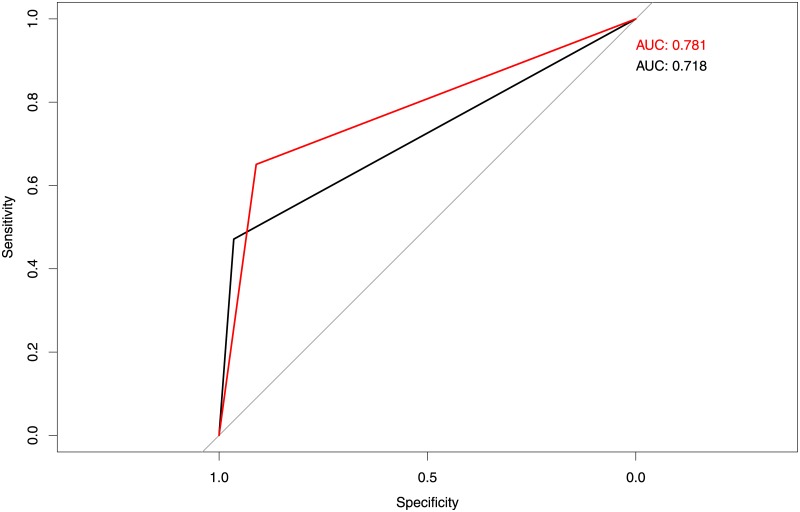
ROC curves and AUC scores derived from the validation of EFFIS standard thresholds (black) and caliver (red) newly calibrated thresholds.

According to EFFIS, the high danger threshold is 21.3 which corresponds to a probability of detection of 47% and an AUC score of 0.718. Repeating the same exercise using caliver’s threshold for Europe (*fire*_*threshold* = 10), the probability of detection increases to 65% and the AUC score to 0.781. Fig 2 shows in black the ROC curve corresponding to EFFIS’ threshold and in red the curve corresponding to caliver’s threshold.

If country-specific danger levels are considered, instead, the high danger threshold fluctuates between 5 and 15. The vignette ‘An introduction to the caliver package’ contains an iteration of the the procedure illustrated above for most of countries in Europe so that hits and misses can be calculated country-by-country, while at the European level hits and misses are re-calculated as the sum over the individual European countries. Table 12 summarises the output of this validation using EFFIS and caliver’s danger levels. Compared to EFFIS, caliver’s methodology returns a systematically higher number of hits and lower number of misses, both at European scale and country level (for United Kingdom, Spain and Italy). Comparing European and country-specific danger levels, the latter provide a further improvement only in northern countries (i.e. UK) while it is generally better to consider European levels for Mediterranean countries. The country-specific danger classes calculated here are only indicative and are obtained without engaging local expertise. To obtain a better estimate at the local scale, we suggest to tune the parameters of this procedure using expert elicitation, for instance to determine a more realistic value for the number of days per year in which a fire is expected to occur.

**Table 12:** Comparison of hits and misses using various danger levels: EFFIS, caliver’s levels over Europe and caliver’s country-specific levels. Caliver’s methodology systematically returns higher number of hits and lower number of misses. The last column shows hits and misses considering Europe as the sum of its parts.

		EFFIS standard-European danger levels	Caliver European danger levels	Caliver country-specific danger levels
**Europe**	Hits	7766	10421	10210
	Misses	8163	5508	5719
**UK**	Hits	4	13	20
	Misses	52	43	36
**Spain**	Hits	1728	2043	1916
	Misses	1019	704	831
**Italy**	Hits	1635	1972	1925
	Misses	907	570	617

## Conclusions

The caliver package is an open source software for the analysis and manipulation of forest fire gridded model results. It is developed in the R statistical language, available from a public repository and distributed under an open licence. This tool enables reproducibility of typical analysis workflows, including calibration and validation/verification. The software was designed and implemented to streamline the processes and based on best practice to reduce processing time and memory resources, hence allow users to rely on accessible hardware. Caliver is currently tailored on the fire forecast of the European Forest Fire Information System (EFFIS) which is produced by the GEFF modeling components, future developments could focus on the generalisation of the internal algorithms so that the same procedures can be used to manipulate the outputs of other types of models, not necessarily related to fire. We invite fellow scientists to contribute to this software and submit issues and suggestions for future developments.

## Supporting information

S1 Appendix Information related to:
Code availability and software requirements.Hardware and software specifications of the system used for testing.
(TEX)
